# Changes of plasma nitric oxide, endothelin-1, and blood coagulation following intravitreal conbercept

**DOI:** 10.1038/s41598-021-03335-3

**Published:** 2021-12-13

**Authors:** Quan-Yong Yi, Li-Shuang Chen, Yu Shen, Yan-Hong Liao, Yan-Yan Wang, Jie Yang, Yuanhui Jin, Lingyun Cheng

**Affiliations:** 1 Ningbo Eye Hospital, Ningbo, China; 2 Institute of Ocular Pharmacology, School of Ophthalmology and Optometry, Wenzhou Medical University, Wenzhou, China; 3 Jacob’s Retina Center at Shiley Eye Institute, Department of Ophthalmology, University of California San Diego, 9415 Campus Point Drive, La Jolla, CA 92037-0946 USA

**Keywords:** Health care, Medical research

## Abstract

Intravitreal anti-VEGF (anti-vascular endothelial growth factor) biologics have revolutionized the pharmacological management of chorioretinal diseases. However, the systemic adverse events such as stroke or bleeding are the concerns for many patients and physicians. The mechanism to develop these side effects are poorly understood. Consecutive 95 patients with retinal diseases were studied for their blood activated partial thromboplastin time (APTT), prothrombin time (PT), international normalized ratio (INR), and concentration of fibrinogen before and after intravitreal conbercept. Additionally, plasma nitric oxide (NO) and endothelin-1 (ET-1) were investigated on 38 of the 95 patients. Compared with the pre-injection, 4-week post-injection values of APTT and PT were increased by 0.582 s (p = 0.038, paired t test) and by 0.086 s (p = 0.080, paired t test; p = 0.0475, Sign test), respectively. At the same time, fibrinogen decreased by 0.048 g/L. Plasma levels of NO or ET-1 or VEGF did not significantly change from pre-injection levels. Our findings advanced the understanding of mechanism for systemic side effects associated with intravitreal anti-VEGF and emphasized paying more attention to higher risk of possible bleedings for patients following intravitreal conbercept.

## Introduction

Since the advent of anti-vascular endothelium growth factor (anti-VEGF) monoclonal antibodies, intravitreal injection of anti-VEGF therapy has proliferated from initial neovascular age-related macular degeneration (nARMD)^1^ to more retinal diseases such as diabetic macular edema (DME)^2^ and retinal vein occlusion (RVO)^3^. Intravitreal anti-VEGF therapy has revolutionized the pharmacological management of those diseases. Intermittent intravitreal anti-VEGF has become a mainstay in management of chorioretinal diseases. In general, ocular use of anti-VEGF appears to be safe for systemic consideration. However, it has been reported that intravitreal injection of anti-VEGF agent may increase the risk of cardiovascular and cerebrovascular events such as myocardial infarction^4^, stroke^5^ and bleeding^6^. In an analysis of intravitreal lucentis for diabetic macular edema, the stroke rate at two years was 2.7% in the patients with intravitreal 0.5 mg lucentis while only 1.1% in the control population (https://www.accessdata.fda.gov/drugsatfda_docs/label/2017/125156s114lbl.pdf). The most recent meta-analysis of systemic adverse events following intravitreal anti-VEGF revealed higher risk of death in diabetic retinopathy patients as well as higher risk of non-ocular bleeding in the patients with age-related macular degeneration^6^. Considering ever growing list of ocular diseases to be treated by local anti-VEGF and explosive number of annual intravitreal anti-VEGF injections, it is imperative to understand the possible mechanism of collateral systemic adverse effects. For the reported systemic adverse events such as blood clogging or bleeding, intuitive reasoning would be looking into blood coagulation parameters. The hypothesis is that intravitreal injected anti-VEGF may enter into the systemic circulation, which might alter the balance of the blood coagulation system. There is hard evidence for the first part of the hypothesis that systemic VEGF can indeed be suppressed by intravitreal injection of anti-VEGF agents^7,8^. For the second part of the hypothesis regarding possible subsequent changes of the blood coagulation system and the underneath mechanism, scientific data is very scarce and heterogeneous in literature. A few studies have investigated blood coagulation parameters after intravitreal injection of aflibercept^9–11^ ranibizumab^12,13^ or bevacizumab^13,14^; however, the results are variable. With low event rate of the systemic adversary and possible drug specific difference or difference of patient condition, a large sample from a real-world practice would be preferable to approach the possible hidden connection between use of intravitreal anti-VEGF and changes of systemic coagulation.

Conbercept (Lumitin^®^, Chengdu Kanghong Biotech Co., Ltd, China) is a recombinant fusion protein composed of the VEGF binding domains of the human VEGFR1 and VEGFR2^15^. It has been approved by the China Food and Drug Regulatory Administration. Numerous clinical trials have shown that intravitreal conbercept has a significant treatment benefit for chorioretinal diseases such as nARMD^16^ and DME^17^. There is no large sample study to assess changes of blood coagulation parameters following intravitreal conbercept. It is reasonable to assume that intravitreal conbercept would have similar systemic side effects as the other intravitreal anti-VEGF agents though these agents do have different molecular sizes and structures as well as VEGF binding affinity^18^. For example, a study has shown that intravitreal conbercept caused more suppression of systemic VEGF than ranibizumab during the first week^19^. The current study aims to investigate change of blood coagulation parameters following the first intravitreal injection of conbercept and the possible changes of nitric oxide (NO) and endothelin-1 (ET-1) in systemic circulation. Both NO and ET-1 are key downstream mediators of the VEGF signaling pathway and the key components maintaining renovascular homeostasis^20–24^. Systemic use of anti-VEGF can induce hypertension and the associated NO suppression as well as ET-1 stimulation^22,25^. We hypothesize that imbalance of NO/ET-1 signaling might be a part of mechanism to develop vascular clogging or bleeding adverse events in addition to changes of coagulation parameters.

## Materials and methods

### Study design

The reported systemic adverse events after intravitreal anti-VEGF are mainly thromboembolic^26^ or bleeding^6^. A logical inference would be that the systemic adverse events may be associated with disfunction of blood coagulation or dysregulation of vascular tone from ocular use of anti-VEGF. Therefore, we were interested in knowing if any changes occurred in blood coagulation parameters or changes in NO and ET-1 plasma levels following intravitreal conbercept. Though the reported changes of coagulation parameters after intravitreal anti-VEGFs are not consistent, hypertension^24^ and the associated changes of NO and ET-1 were reported following systemic use of anti-VEGF agents^25,27–29^. NO is a potent vasodilator while ET-1 is a potent vasoconstrictor. Both NO and ET-1 are endothelium-specific and the major regulators of blood pressure (BP)^30^. Hypertension is a known risk factor for triggering thromboembolic events and hemorrhagic events.

For the current study, blood coagulation parameters of 95 patients were analyzed by comparing before and 4-week after the first intravitreal conbercept (prior to the 2nd intravitreal conbercept). Blood coagulation test is a routine for use of anti-VEGF biologics in this institute; therefore, all 95 patients had the coagulation parameters available. Out of 95 patients, 38 patients agreed for additional blood draw to quantitate NO, ET-1, and VEGF in plasma. In order to better delineate the possible relationship between intravitreal anti-VEGF and change of systemic coagulation parameters, patients with diabetic macular edema and retinal vein occlusion were not included. It is known that both advanced diabetes and vein occlusive diseases have abnormal platelet function and coagulation^31,32^. This study was performed at Ningbo Eye Hospital at Zhejiang province of China and the study was approved by the ethics committee of the Ningbo Eye Hospital (qtky-006). The study adhered to the tenets of the declaration of Helsinki.

### Patients and setting

Ninety-five consecutive patients between February 24 of 2018 and December 7 of 2019 participated in this study. The including criteria are: (1) retinal patients naïve to intravitreal anti-VEGF; (2) no history of local or systemic steroid usage for at least four months prior to the current visit; (3) patients were excluded with history of heart attack or stroke or taking antiplatelet agents or warfarin medication. Written informed consent was provided to all the patients; and treatment benefits as well as possible risks were well explained. The blood pressures were recorded and blood samples were collected via left arm cephalic vein one day before the intravitreal injection. A comprehensive ophthalmic exam was performed on the same day of intravitreal injection. All patients had blood coagulation parameters tested while 38 patients had additional quantitation of NO, ET-1, and VEGF in plasma. All patients were instructed to report any noted changes in blood pressure or adverse events after intravitreal conbercept.

### Blood pressure measurement

The blood pressures were measured a day before the intravitreal injection, and the blood sampling was 30 min after the blood pressure was taken. All blood measurement was performed with a same unit (an automated sphygmomanometer, HEM-7120, Omron Healthcare, Tokyo, Japan) for better consistency. Patients were asked to take a seat for 5 min resting before the blood pressure was taken. To minimize the effect of diurnal variation of blood pressure, all pressures were taken between 8 and 10 am.

### Intravitreal injection

In this hospital, intravitreal injection was required to be performed in an ophthalmic surgery suite. The eyeball surface was numbed by 0.5% proparacaine eye drops. After the eyeball rinsed with balanced salt solution (BSS), a drop of 5% povidone iodine was instilled for disinfection. Conbercept 0.5 mg in 50 µL was injected into the vitreous cavity from 3.5 mm behind the limbus at the infratemporal quadrant using a 30-gauge needle attached to a 1 cc syringe. 0.5% levofloxacin eye drops three time daily for 5 days was prescribed for preventative measure.

### Coagulation function test

Blood was collected into an anti-coagulation tube containing citric acid (citric volume to blood volume ratio of 1:9) and the tube was centrifugated with a relative centrifugal force of 2862g at 4 °C for 10 min using a low-speed centrifuge (KDC-40, Zonkia scientific instruments Co., LTD, Anhui, China). The aspirated plasma was tested for prothrombin time (PT), activated partial thromboplastin time (APTT), international normalized ratio (INR) and fibrinogen using an automatic coagulation analyzer (CA-1500, Sysmex, Tokyo, Japan, with its auxiliary reagents: PT assay kit, OUHP29, Sysmex, Tokyo, Japan; APTT assay kit, B4218-1, Sysmex, Tokyo, Japan; Fibrinogen assay kit, B4233-27, Sysmex, Tokyo, Japan).

The international normalized ratio (INR) was calculated using the following formula:

$$INR={\left(\frac{Patient's PT}{MNPT}\right)}^{ISI}$$

where MNPT stand for the mean normal prothrombin time and ISI stand for the international sensitivity index which is a parameter of the PT assay kit and was calibrated and provided by the manufacturer.

1. PT: excess tissue thromboplastin (tissue factor) was added to the plasma samples, and with the participation of Ca^2+^, prothrombin was converted to thrombin and fibrinogen was converted to insoluble fibrin. PT was recorded as the time required for samples to coagulate.
2. APTT: the ellagic acid was added to the plasma samples under 37 °C water bath, and with the participation of Ca^2+^, the endogenous coagulation system was activated and fibrinogen was converted to insoluble fibrin. APTT was recorded as the time required for samples to coagulate.
3. Fibrinogen: excess thrombin was added to the plasma samples, and fibrinogen was converted to insoluble fibrin. The time required for samples to coagulate was negatively correlated with the concentration of fibrin and a standard curve was constructed to determine the concentration of fibrinogen.

### Quantification of nitric oxide (NO), endothelin-1 (ET-1) and VEGF

The aspirated plasma was kept under -80 °C until the analysis. Plasma levels of NO were quantitated by measuring NO metabolites (nitrates and nitrites) using a commercially available kit according to manufacturer’s instruction (Parameter™ Total Nitric Oxide and Nitrate/Nitrite Assay, KGE001, R&D Systems, Inc., Minneapolis, MN, USA). This assay determined NO concentrations based on the enzymatic conversion of nitrate to nitrite by nitrate reductase. The reaction was followed by colorimetric detection of nitrite as an azo dye product of the Griess Reaction. A standard curve was constructed from 3.13 to 200 μmol/L by four parameter logistic (4-PL) curve-fit. The lower limit of quantitation (LLOQ) for NO was 0.25 μmol/L.

Plasma levels of ET-1 and VEGF were quantitated by commercially available kits according to manufacturer’s instruction (Human ET-1 ELISA Kit, CSB-E07007h, CUSABIO, Inc., Wuhan, China; Human VEGF ELISA Kit, CSB-E11718h, CUSABIO, Inc., Wuhan, China). The assays determined ET-1 and VEGF concentrations based on the principle of sandwich ELISA. A standard curve was constructed from 3.12 to 200 pg/mL for ET-1 and 31.25 to 2000 pg/mL for VEGF by 4-PL curve-fit. The LLOQ was 0.78 pg/mL for ET-1 and 10.122 pg/mL for VEGF.

### Statistical analysis

Entities of eye diseases and systemic diseases as well as demographic characteristics of the patients were tabulated with means ± standard deviations for continuous data and counts or fractions for binary data. The changes of blood pressures or blood coagulation parameters between pre- and post-intravitreal conbercept were assessed by paired t-test while the changes of vasotonic regulators were assessed by signed rank test. JMP statistical software was used for all analysis (JMP^®^, Version < 13 >  SAS Institute Inc., Cary, NC, 1989–2007).

## Results

### Characteristics of the participants

Ninety-five patients participated in this study. Of those, 38 patients had quantitation of plasma vasotonic regulators (NO, ET-1, and VEGF). Eye diseases distribution and the accompanying systemic diseases along with status of systemic medication were summarized in Table 1. The majority of accompanying systemic diseases are high blood pressure and mild diabetes without diabetic macular edema.

**Table 1:** Demographic and clinical features of the patients.

Eye diseases	Cases	Age	Gender	Systemic diseases (#)	Systemic medication	Vasotonic regulators tested (Y, N)
nAMD	59	67.44 ± 10.22	M37/f22	NS(25)/HP(22)/DB(8)/O(4)	Y(28)/N(31)	Y(22)/N(37)
PCV	19	67.11 ± 7.43	m13/f6	NS(7)/HP(6)/DB(5)/O(1)	Y(12)/N(7)	Y(7)/N(12)
PM-CNV	17	54.53 ± 15.84	m1/f16	NS(9)/HP(5)/DB(2)/O(1)	Y(8)/N(9)	Y(9)/N(8)

*nAMD* neovascular age-related macular degeneration, *PCV* polypoidal choroidal vasculopathy, *PM-CNV* pathologic myopic choroidal neovascularization, *DB* diabetes, *HP* high blood pressure, *NS* no identified systemic disease, *O* others (lipidemia, Parkinson’s disease, and vitiligo).

### Blood coagulation parameters

**Figure 1:**
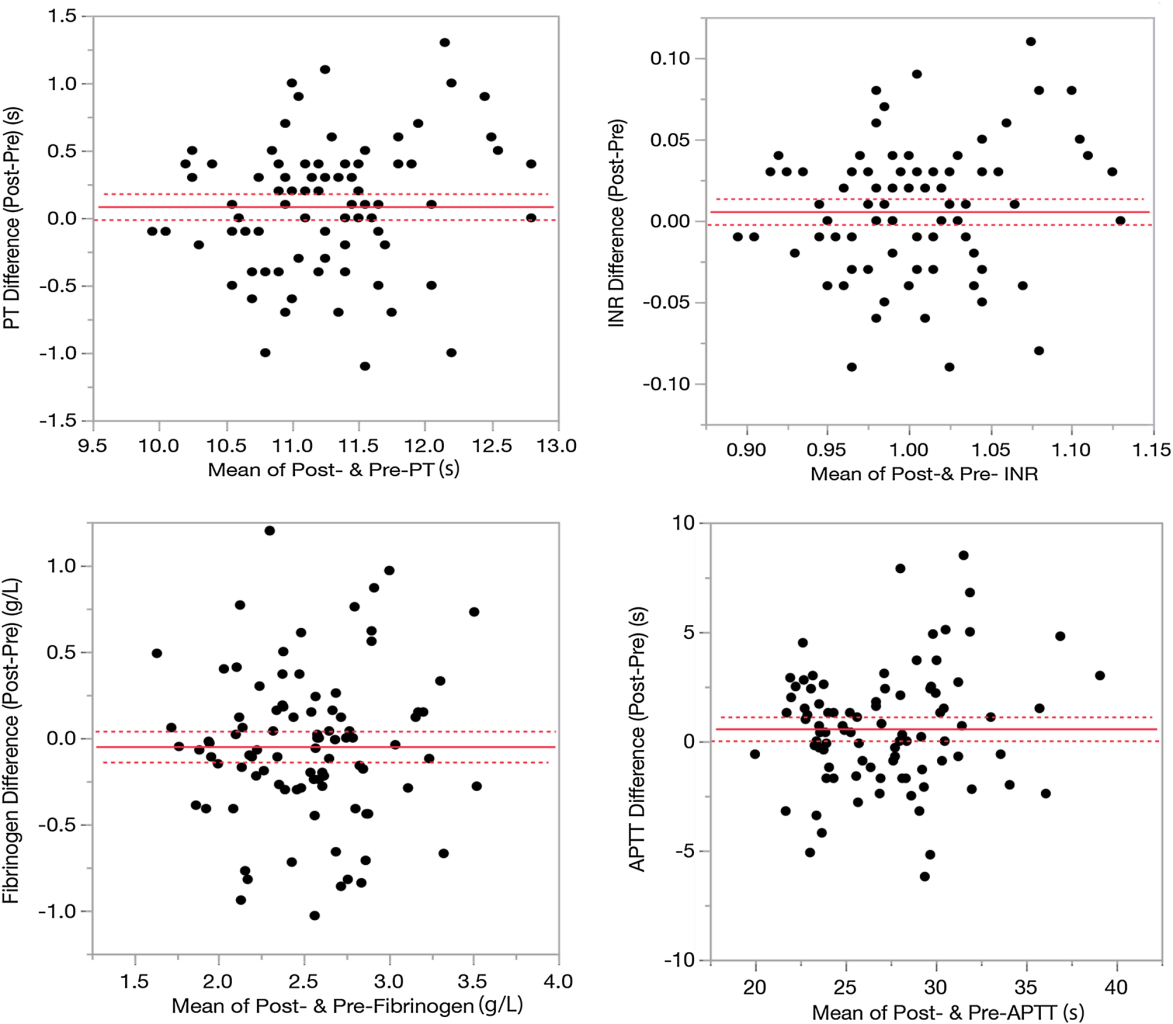
The differences plots of prothrombin time (PT, upper left), international normalized ratio (INR, upper right), fibrinogen (lower left), and activated partial thromboplastin time (APTT, lower right). The solid horizontal line in each frame represents the mean difference and the two dotted lines, encompassing the mean difference line, represent the 95% confidence interval.

The differences between pre- and post-intravitreal conbercept for PT, INR, Fibrinogen, and APTT were plotted in Fig. 1. PT increased by 0.086 s from pre intravitreal injection (p = 0.080, paired t-test; = 0.0475, Sign test). Similarly, APTT and INR increased by 0.582 s (p = 0.038) and 0.006 s (p = 0.153), respectively. Fibrinogen was decreased by 0.048 g/L (p = 0.286). Significant PT and APTT increase and the trend of Fibrinogen decrease may indicate an early imbalance of the coagulation system, which may be leading to a tendency of bleeding.

### The reported events, blood pressure, and vasotonic regulators changes

Between the first intravitreal and second intravitreal injection, two patients reported the noted BP elevation and corresponding dose increase of anti HP drugs. No other adverse events were reported. Blood pressures at mean 31 days after the intravitreal injection of conbercept were systolic 131 ± 15 mmHg/diastolic 77 ± 9 mmHg which did not change significantly from pre-intravitreal injection systolic 133 ± 18 mmHg/diastolic 78 ± 10 mmHg. NO and ET-1 did not change significantly from pre intravitreal injection (NO pre 78.03 ± 95.80 vs. post 92.08 ± 81.53 µmol/L, p = 0.21, signed-rank test; ET-1 pre 3.54 ± 1.66 vs. post 3.63 ± 1.65 pg/mL, p = 0.70, signed-rank test). For VEGF in plasma, there was no difference either between pre (17.45 ± 13.75 pg/mL) and post intravitreal conbercept (16.65 ± 15.86 pg/mL).

### Systemic diseases and conbercept-induced parameters change

The changes of blood coagulation parameters and vasotonic regulators (NO and ET-1) were assessed over systemic disease types in regression analyses while adjusting for age and gender. No significant association was found between the parameter changes and types of systemic disease.

## Discussion

Systemic safety of intravitreal anti-VEGF is still a debate depending on the viewpoints^33,34^. However, there are hard evidences that free VEGF levels in plasma were decreased even 4 weeks after intravitreal bevacizumab, ranibizumab, and aflibercept^7^. Currently the mechanism for developing adverse events such as stroke or bleeding is not clear. We hypothesize that the causes may be multifaceted including specific drug related blood chemistry changes and patients’ health conditions. A few studies have looked into the blood coagulation parameters following intravitreal injection of bevacizumab^14^ or aflibercept^9,10^ or ranibizumab^12^. In contrast, so far there is no similar report following intravitreal conbercept. The current study revealed that blood coagulation parameters moved towards longer time to clot or less clotting after intravitreal conbercept. APPT was extended by 0.58 s which is statistically significant. Similarly, PT was also extended by 0.09 s with a p value of 0.0475 (Sign test) (0.08 for two tails test or 0.04 for one tail test if use paired t-test). Though the changes of fibrinogen from pre intravitreal conbercept was not statistically significant, the direction of the change was supportive to the observed increase of APPT and PT. Current findings are from a large sample (n = 95) of ophthalmic clinic patients derived from a real-world practice. In contrary to the reports after intravitreal bevacizumab (n = 60) or aflibercept (n = 34) which both led to decreased PT^14^ and APPT^9^. Increased blood clotting would be logical to interpret the observed adverse event such as stroke; however, nonocular hemorrhagic events in patients treated with intravitreal ranibizumab was reported to be significantly more than in control trials^6^. In addition to pre health conditions of patients sampled, specific anti-VEGF agent may also contribute to the observed differences from these studies. For example, several well-designed studies have demonstrated that drop of plasma VEGF after intravitreal anti-VEGFs were much greater for bevacizumab then for aflibercept and the least for ranibizumab due to possible larger molecular size with longer plasma half-life^18,35^. In effect, how these anti-VEGF agents affect the systemic vascular system and cause possible collateral adverse events may be much more complicated than we are thinking of. The process may be a multifaceted, including patients’ predisposition, specific binding capacity of these agents to host endothelial cells and possible resultant inflammation^36^, as well as acute vascular tonicity change and blood pressure flare-ups via changes of blood vessel vasotonic regulation^37^. Bevacizumab, ranibizumab and aflibercept came into clinical practice earlier and better studied for their systemic adverse events. For conbercept, there is very little information available about its systemic adverse events following intravitreal injection.

To better understand the mechanism for systemic vascular change after intravitreal conbercept, we quantitated levels of NO and ET-1, two key vasoactive factors, in plasma before and after intravitreal injection. Hypertension is a well-known risk for cardiac and cerebral stroke. We hypothesize that conbercept leaked to systemic circulation from the eye may cause decreased NO and increased ET-1 in plasma which may contribute to elevation of blood pressure. Systemically used anti-VEGF agents have shown decreased NO^29^ and increased ET-1^27^ along with hypertension^25^. ET-1 increase in plasma has also been reported in bevacizumab-treated retinopathy of prematurity patients^38^. Current study did not reveal the significant mean blood pressures elevation or significant change of plasma NO or ET-1 from pre intravitreal conbercept though two patients reported brief elevation of blood pressures. Brief elevation of blood pressure is hard to capture but can act as a trigger for adverse vascular events. It is possible that intravitreal conbercept-induced plasma changes of NO and ET-1 was transient and outside of our blood sampling time frame. In this regard, more blood sampling time points would be preferable if the research ethic is met and with patient consent.

Structurally, conbercept is more similar to aflibercept than to ranibizumab because both are fusion protein of human VEGF receptors with IgG-Fc component. A most recent prospective interventional study did reveal similar findings to the current study that significant prolongation of thrombin time and strong trend of increased APTT two to three days following intravitreal aflibercept^36^. In the current study, increased PT and APTT were still detected 4 weeks after the initial intravitreal injection of conbercept. Considering low incidence of the systemic adverse event in real world practice, meta-analysis may offer more dependable information. A most recent meta-analysis from JAMA Ophthalmology revealed that administration of anti-VEGF agents increased the risk of non-ocular hemorrhage in AMD patients (OR = 1.46, 95% CI 1.01–2.10); as well as increased risk of death in patients with diabetic retinopathy (OR = 1.80, 95% CI 1.03–3.16)^39^.

In summary, current study revealed increased blood coagulation time even a month after intravitreal conbercept, which was not reported in literature. This finding needs to be validated by more similar studies. The observed changes may or may not contribute to systemic adverse events because these parameters are still in the normal range; however, patients receiving intravitreal conbercept should be observed for bleeding or encouraged to report their own observations such as easy bruisedness or bleeding elsewhere. Further studies are needed to address when the blood coagulation parameters would return to baseline following a single intravitreal injection or how much further changes of the parameters would be if multiple intravitreal injections were performed. No empirical blood pressure elevations or vasoactive factors were detected by the current study; however, blood pressure elevation and vasoactive NO and ET-1 changes at earlier time point following intravitreal conbercept cannot be ruled out.
